# 2,4-Dimethyl-*N*-(4-methyl­phen­yl)benzene­sulfonamide

**DOI:** 10.1107/S1600536810049627

**Published:** 2010-12-04

**Authors:** P. G. Nirmala, Sabine Foro, B. Thimme Gowda

**Affiliations:** a Department of Chemistry, Mangalore University, Mangalagangotri 574 199, Mangalore, India; b Institute of Materials Science, Darmstadt University of Technology, Petersenstrasse 23, D-64287 Darmstadt, Germany

## Abstract

The asymmetric unit of the crystal of the title compound, C_15_H_17_NO_2_S, contains two independent mol­ecules, which are twisted at the S—N bonds with C—SO_2_—NH—C torsion angles of 48.3 (2) (mol­ecule 1) and −75.7 (3)° (mol­ecule 2). The dihedral angles between the benzene rings are 72.0 (1) (mol­ecule 1) and 78.3 (1)° (mol­ecule 2). The crystal structure features inversion dimers linked by pairs of N—H⋯O hydrogen bonds.

## Related literature

For the preparation of the title compound, see: Savitha & Gowda (2006 ▶). For our studies of the effect of substituents on the structures of *N*-(ar­yl)aryl­sulfonamides, see: Gowda *et al.* (2009 ▶); Nirmala *et al.* (2009 ▶, 2010 ▶). For related structures, see: Gelbrich *et al.* (2007 ▶); Perlovich *et al.* (2006 ▶).
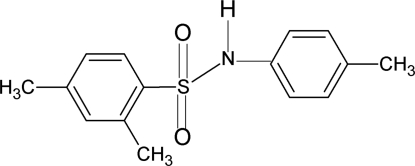


## Experimental

### 

#### Crystal data

C_15_H_17_NO_2_S
*M* *_r_* = 275.36
Monoclinic, 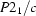

*a* = 10.623 (1) Å
*b* = 10.770 (1) Å
*c* = 25.513 (2) Å
β = 97.927 (6)°
*V* = 2891.0 (4) Å^3^
*Z* = 8
Mo *K*α radiation
μ = 0.22 mm^−1^
*T* = 299 K
0.40 × 0.30 × 0.20 mm

#### Data collection

Oxford Diffraction Xcalibur diffractometer with a Sapphire CCD detector
Absorption correction: multi-scan (*CrysAlis RED*; Oxford Diffraction, 2009 ▶) *T*_min_ = 0.917, *T*_max_ = 0.957
10876 measured reflections
5282 independent reflections
3743 reflections with *I* > 2σ(*I*)
*R*_int_ = 0.017

#### Refinement

*R*[*F* ^2^ > 2σ(*F* ^2^)] = 0.041
*wR*(*F* ^2^) = 0.116
*S* = 1.03
5282 reflections
355 parameters
3 restraints
H atoms treated by a mixture of independent and constrained refinement
Δρ_max_ = 0.20 e Å^−3^
Δρ_min_ = −0.23 e Å^−3^

### 

Data collection: *CrysAlis CCD* (Oxford Diffraction, 2009 ▶); cell refinement: *CrysAlis RED* (Oxford Diffraction, 2009 ▶); data reduction: *CrysAlis RED*; program(s) used to solve structure: *SHELXS97* (Sheldrick, 2008 ▶); program(s) used to refine structure: *SHELXL97* (Sheldrick, 2008 ▶); molecular graphics: *PLATON* (Spek, 2009 ▶); software used to prepare material for publication: *SHELXL97*.

## Supplementary Material

Crystal structure: contains datablocks I, global. DOI: 10.1107/S1600536810049627/bq2257sup1.cif

Structure factors: contains datablocks I. DOI: 10.1107/S1600536810049627/bq2257Isup2.hkl

Additional supplementary materials:  crystallographic information; 3D view; checkCIF report

## Figures and Tables

**Table 1:** Hydrogen-bond geometry (Å, °)

*D*—H⋯*A*	*D*—H	H⋯*A*	*D*⋯*A*	*D*—H⋯*A*
N1—H1*N*⋯O3^i^	0.85 (1)	2.15 (1)	2.991 (2)	169 (2)
N2—H2*N*⋯O2^ii^	0.85 (1)	2.03 (1)	2.877 (2)	175 (3)

Symmetry codes: (i) 
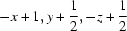
; (ii) 
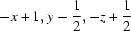
.

**Fig. 1.**
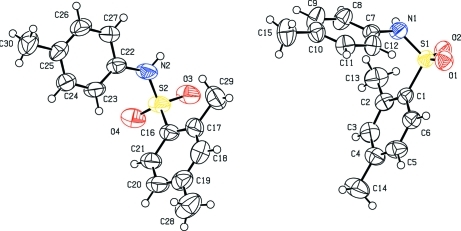
Molecular structure of (I), showing the atom labeling scheme and displacement ellipsoids are drawn at the 50% probability level.

**Fig. 2.**
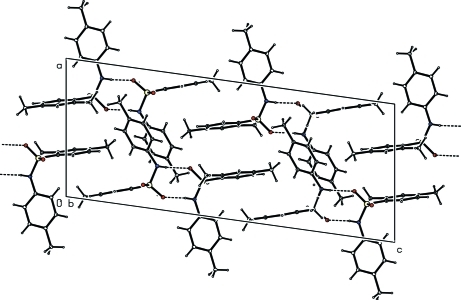
Molecular packing of (I) with hydrogen bonding shown as dashed lines.

## supplementary crystallographic information

### Comment

As part of a study of the effect of substitutions on the structures of *N*-(aryl)-arylsulfonamides (Gowda *et al.* , 2009; Nirmala *et al.*, 2009; 2010), in the present work, the structure of 2,4-dimethyl-*N*-(4-methylphenyl)benzenesulfonamide (I) has been determined (Fig. 1). The asymmetric unit of (I) contains two independent molecules. The molecules are twisted at the S—N bonds with the C1—SO_2_—NH—C7 torsion angles of -48.3 (2)° (molecule 1) and -75.7 (3)° (molecule 2), compared to the values of 71.6 (1)° in 2,4-dimethyl-*N*-(2-methylphenyl)benzenesulfonamide (II) (Nirmala *et al.*, 2009), -58.4 (2)° in 2,4-dimethyl-*N*-(3-methylphenyl)benzenesulfonamide (III) (Nirmala *et al.*, 2010) and -46.1 (3)° (molecule 1) & 47.7 (3)° (molecule 2) in the two molecules of 2,4-dimethyl-*N*-(phenyl)- benzenesulfonamide (IV)(Gowda *et al.*, 2009).

The sulfonyl benzene and the aniline benzene rings in (I) are tilted relative to each other by 72.0 (1)° (molecule 1) and 78.3 (1)° (molecule 2), compared to the values of 47.0 (1)° in (II), 47.1 (1)° in (III), and 67.5 (1)° in molecule 1 and 72.9 (1)° in molecule 2 of (IV).

The other bond parameters in (I) are similar to those observed in (II), (III), (IV) and other aryl sulfonamides (Perlovich *et al.*, 2006; Gelbrich *et al.*, 2007). The crystal packing of molecules in (I) *via* N—H···O(S) hydrogen bonds (Table 1) is shown in Fig.2.

### Experimental

The solution of 1,3-xylene (1,3-dimethylbenzene) (10 ml) in chloroform (40 ml) was treated dropwise with chlorosulfonic acid (25 ml) at 0° C. After the initial evolution of hydrogen chloride subsided, the reaction mixture was brought to room temperature and poured into crushed ice in a beaker. The chloroform layer was separated, washed with cold water and allowed to evaporate slowly. The residual 2,4-dimethylbenzenesulfonylchloride was treated with *p*-toluidine in the stoichiometric ratio and boiled for ten minutes. The reaction mixture was then cooled to room temperature and added to ice cold water (100 ml). The resultant solid 2,4-dimethyl-*N*-(4-methylphenyl)benzenesulfonamide was filtered under suction and washed thoroughly with cold water. It was then recrystallized to constant melting point from dilute ethanol. The purity of the compound was checked and characterized by recording its infrared and NMR spectra (Savitha & Gowda, 2006).

The prism like colourless single crystals used in x-ray diffraction studies were grown in ethanolic solution by a slow evaporation at room temperature.

### Refinement

The H atoms of the NH groups were located in a difference map and later restrained to N—H = 0.86 (1) Å. The other H atoms were positioned with idealized geometry using a riding model with C—H = 0.93–0.96 Å. All H atoms were refined with isotropic displacement parameters (set to 1.2 times of the *U*_eq_ of the parent atom).

### Crystal data

**Table:**

C_15_H_17_NO_2_S	*F*(000) = 1168
*M**_r_* = 275.36	*D*_x_ = 1.265 Mg m^−^^3^
Monoclinic, *P*2_1_/*c*	Mo *K*α radiation, λ = 0.71073 Å
Hall symbol: -P 2ybc	Cell parameters from 2957 reflections
*a* = 10.623 (1) Å	θ = 2.7–27.9°
*b* = 10.770 (1) Å	µ = 0.22 mm^−^^1^
*c* = 25.513 (2) Å	*T* = 299 K
β = 97.927 (6)°	Prism, colourless
*V* = 2891.0 (4) Å^3^	0.40 × 0.30 × 0.20 mm
*Z* = 8	

### Data collection

**Table:**

Oxford Diffraction Xcalibur diffractometer with a Sapphire CCD detector	5282 independent reflections
Radiation source: fine-focus sealed tube	3743 reflections with *I* > 2σ(*I*)
graphite	*R*_int_ = 0.017
Rotation method data acquisition using ω and phi scans	θ_max_ = 25.4°, θ_min_ = 3.1°
Absorption correction: multi-scan (*CrysAlis RED*; Oxford Diffraction, 2009)	*h* = −12→10
*T*_min_ = 0.917, *T*_max_ = 0.957	*k* = −12→11
10876 measured reflections	*l* = −26→30

### Refinement

**Table:**

Refinement on *F*^2^	Primary atom site location: structure-invariant direct methods
Least-squares matrix: full	Secondary atom site location: difference Fourier map
*R*[*F*^2^ > 2σ(*F*^2^)] = 0.041	Hydrogen site location: inferred from neighbouring sites
*wR*(*F*^2^) = 0.116	H atoms treated by a mixture of independent and constrained refinement
*S* = 1.03	*w* = 1/[σ^2^(*F*_o_^2^) + (0.0588*P*)^2^ + 0.5667*P*] where *P* = (*F*_o_^2^ + 2*F*_c_^2^)/3
5282 reflections	(Δ/σ)_max_ = 0.009
355 parameters	Δρ_max_ = 0.20 e Å^−^^3^
3 restraints	Δρ_min_ = −0.23 e Å^−^^3^

### Special details

**Table:**

Experimental. CrysAlis RED (Oxford Diffraction, 2009) Empirical absorption correction using spherical harmonics, implemented in SCALE3 ABSPACK scaling algorithm.
Geometry. All e.s.d.'s (except the e.s.d. in the dihedral angle between two l.s. planes) are estimated using the full covariance matrix. The cell e.s.d.'s are taken into account individually in the estimation of e.s.d.'s in distances, angles and torsion angles; correlations between e.s.d.'s in cell parameters are only used when they are defined by crystal symmetry. An approximate (isotropic) treatment of cell e.s.d.'s is used for estimating e.s.d.'s involving l.s. planes.
Refinement. Refinement of *F*^2^ against ALL reflections. The weighted *R*-factor *wR* and goodness of fit *S* are based on *F*^2^, conventional *R*-factors *R* are based on *F*, with *F* set to zero for negative *F*^2^. The threshold expression of *F*^2^ > σ(*F*^2^) is used only for calculating *R*-factors(gt) *etc*. and is not relevant to the choice of reflections for refinement. *R*-factors based on *F*^2^ are statistically about twice as large as those based on *F*, and *R*- factors based on ALL data will be even larger.

### Fractional atomic coordinates and isotropic or equivalent isotropic displacement parameters (Å^2^)

**Table:**

	*x*	*y*	*z*	*U*_iso_*/*U*_eq_	
S1	0.16582 (5)	0.91378 (5)	0.253220 (19)	0.04951 (16)	
O1	0.16198 (14)	1.03883 (14)	0.23457 (6)	0.0619 (4)	
O2	0.09258 (13)	0.88329 (15)	0.29500 (5)	0.0625 (4)	
N1	0.31153 (16)	0.87937 (18)	0.27751 (6)	0.0543 (5)	
H1N	0.311 (2)	0.8300 (18)	0.3034 (7)	0.065*	
C1	0.12183 (16)	0.81649 (19)	0.19825 (7)	0.0437 (5)	
C2	0.12013 (18)	0.6871 (2)	0.20238 (8)	0.0520 (5)	
C3	0.0833 (2)	0.6212 (2)	0.15577 (9)	0.0616 (6)	
H3	0.0810	0.5350	0.1576	0.074*	
C4	0.0500 (2)	0.6771 (2)	0.10694 (9)	0.0615 (6)	
C5	0.0507 (2)	0.8053 (3)	0.10508 (8)	0.0639 (6)	
H5	0.0264	0.8452	0.0729	0.077*	
C6	0.08644 (19)	0.8752 (2)	0.14974 (8)	0.0538 (5)	
H6	0.0870	0.9614	0.1476	0.065*	
C7	0.41385 (18)	0.8671 (2)	0.24720 (7)	0.0472 (5)	
C8	0.5031 (2)	0.7771 (2)	0.26186 (9)	0.0666 (6)	
H8	0.4941	0.7249	0.2901	0.080*	
C9	0.6063 (2)	0.7634 (3)	0.23492 (11)	0.0761 (7)	
H9	0.6663	0.7025	0.2458	0.091*	
C10	0.6230 (2)	0.8373 (2)	0.19238 (9)	0.0612 (6)	
C11	0.5324 (2)	0.9270 (2)	0.17813 (9)	0.0611 (6)	
H11	0.5413	0.9788	0.1497	0.073*	
C12	0.4286 (2)	0.9428 (2)	0.20474 (9)	0.0582 (6)	
H12	0.3689	1.0041	0.1941	0.070*	
C13	0.1564 (3)	0.6158 (2)	0.25302 (10)	0.0769 (7)	
H13A	0.1038	0.6417	0.2787	0.092*	
H13B	0.2439	0.6316	0.2663	0.092*	
H13C	0.1446	0.5286	0.2463	0.092*	
C14	0.0177 (3)	0.5997 (3)	0.05741 (10)	0.0884 (9)	
H14A	0.0130	0.5138	0.0669	0.106*	
H14B	0.0824	0.6104	0.0350	0.106*	
H14C	−0.0627	0.6258	0.0389	0.106*	
C15	0.7355 (3)	0.8194 (3)	0.16271 (12)	0.0923 (9)	
H15A	0.7062	0.7916	0.1274	0.111*	
H15B	0.7919	0.7585	0.1806	0.111*	
H15C	0.7798	0.8968	0.1613	0.111*	
S2	0.75114 (5)	0.19437 (7)	0.08416 (2)	0.0658 (2)	
O3	0.67144 (15)	0.23202 (19)	0.12238 (6)	0.0836 (6)	
O4	0.75504 (17)	0.06565 (18)	0.07007 (6)	0.0795 (5)	
N2	0.89188 (18)	0.2396 (2)	0.10928 (7)	0.0728 (6)	
H2N	0.892 (2)	0.284 (2)	0.1371 (7)	0.087*	
C16	0.70689 (18)	0.2795 (2)	0.02500 (8)	0.0569 (6)	
C17	0.6886 (2)	0.4078 (3)	0.02402 (10)	0.0677 (7)	
C18	0.6524 (2)	0.4629 (3)	−0.02539 (12)	0.0800 (8)	
H18	0.6401	0.5484	−0.0268	0.096*	
C19	0.6339 (2)	0.3966 (3)	−0.07224 (11)	0.0761 (7)	
C20	0.6518 (2)	0.2702 (3)	−0.06978 (9)	0.0737 (7)	
H20	0.6392	0.2236	−0.1007	0.088*	
C21	0.6883 (2)	0.2111 (3)	−0.02178 (8)	0.0647 (6)	
H21	0.7003	0.1256	−0.0208	0.078*	
C22	1.00993 (19)	0.2099 (2)	0.09259 (8)	0.0547 (5)	
C23	1.0219 (2)	0.1542 (3)	0.04490 (8)	0.0709 (7)	
H23	0.9497	0.1309	0.0222	0.085*	
C24	1.1411 (2)	0.1330 (3)	0.03075 (9)	0.0716 (7)	
H24	1.1473	0.0951	−0.0016	0.086*	
C25	1.2500 (2)	0.1658 (2)	0.06267 (11)	0.0655 (6)	
C26	1.2360 (2)	0.2188 (2)	0.11096 (11)	0.0740 (7)	
H26	1.3085	0.2403	0.1339	0.089*	
C27	1.1183 (2)	0.2407 (2)	0.12624 (9)	0.0626 (6)	
H27	1.1121	0.2761	0.1590	0.075*	
C28	0.5989 (3)	0.4606 (4)	−0.12494 (13)	0.1115 (11)	
H28A	0.6747	0.4790	−0.1400	0.134*	
H28B	0.5545	0.5363	−0.1199	0.134*	
H28C	0.5452	0.4071	−0.1484	0.134*	
C29	0.7054 (3)	0.4885 (3)	0.07303 (12)	0.0962 (9)	
H29A	0.7878	0.4738	0.0927	0.115*	
H29B	0.6410	0.4686	0.0946	0.115*	
H29C	0.6982	0.5743	0.0629	0.115*	
C30	1.3795 (2)	0.1458 (3)	0.04557 (14)	0.0971 (10)	
H30A	1.3696	0.1275	0.0084	0.117*	
H30B	1.4212	0.0776	0.0650	0.117*	
H30C	1.4297	0.2196	0.0525	0.117*	

### Atomic displacement parameters (Å^2^)

**Table:**

	*U*^11^	*U*^22^	*U*^33^	*U*^12^	*U*^13^	*U*^23^
S1	0.0446 (3)	0.0605 (4)	0.0442 (3)	0.0057 (2)	0.0088 (2)	0.0021 (2)
O1	0.0653 (9)	0.0528 (10)	0.0674 (9)	0.0038 (7)	0.0093 (7)	0.0006 (8)
O2	0.0517 (8)	0.0895 (12)	0.0494 (8)	0.0140 (8)	0.0179 (7)	0.0080 (8)
N1	0.0448 (9)	0.0755 (13)	0.0429 (9)	0.0036 (9)	0.0073 (8)	0.0052 (9)
C1	0.0367 (10)	0.0514 (13)	0.0438 (10)	0.0015 (8)	0.0084 (8)	0.0062 (9)
C2	0.0432 (11)	0.0571 (14)	0.0561 (12)	−0.0066 (9)	0.0086 (9)	0.0082 (11)
C3	0.0567 (13)	0.0556 (14)	0.0742 (15)	−0.0129 (10)	0.0148 (11)	−0.0024 (12)
C4	0.0527 (13)	0.0767 (18)	0.0567 (13)	−0.0161 (12)	0.0129 (10)	−0.0102 (12)
C5	0.0650 (14)	0.0832 (19)	0.0432 (12)	−0.0065 (12)	0.0069 (10)	0.0070 (12)
C6	0.0560 (12)	0.0585 (14)	0.0472 (11)	0.0020 (10)	0.0088 (9)	0.0088 (10)
C7	0.0419 (10)	0.0580 (13)	0.0412 (10)	−0.0027 (9)	0.0037 (8)	−0.0031 (9)
C8	0.0546 (13)	0.0815 (17)	0.0654 (14)	0.0116 (12)	0.0146 (11)	0.0223 (13)
C9	0.0589 (15)	0.0785 (18)	0.0937 (19)	0.0209 (12)	0.0208 (13)	0.0210 (15)
C10	0.0551 (13)	0.0620 (15)	0.0700 (14)	−0.0017 (11)	0.0209 (11)	−0.0031 (12)
C11	0.0640 (14)	0.0621 (15)	0.0606 (13)	−0.0046 (12)	0.0203 (11)	0.0076 (12)
C12	0.0543 (13)	0.0565 (14)	0.0655 (14)	0.0045 (10)	0.0137 (10)	0.0054 (11)
C13	0.0869 (18)	0.0626 (17)	0.0778 (17)	−0.0075 (13)	−0.0017 (14)	0.0199 (13)
C14	0.0858 (19)	0.109 (2)	0.0728 (17)	−0.0318 (16)	0.0184 (14)	−0.0272 (16)
C15	0.0802 (18)	0.095 (2)	0.112 (2)	0.0100 (16)	0.0499 (17)	0.0032 (18)
S2	0.0534 (3)	0.1010 (5)	0.0443 (3)	−0.0202 (3)	0.0115 (2)	−0.0084 (3)
O3	0.0596 (10)	0.1410 (17)	0.0543 (9)	−0.0271 (10)	0.0221 (7)	−0.0182 (10)
O4	0.0850 (11)	0.0914 (9)	0.0616 (10)	−0.0235 (9)	0.0085 (8)	0.0003 (9)
N2	0.0517 (11)	0.1221 (19)	0.0448 (10)	−0.0140 (11)	0.0076 (8)	−0.0212 (11)
C16	0.0397 (11)	0.0814 (18)	0.0505 (12)	−0.0113 (10)	0.0089 (9)	−0.0098 (11)
C17	0.0463 (12)	0.088 (2)	0.0709 (16)	−0.0035 (12)	0.0175 (11)	−0.0183 (14)
C18	0.0618 (15)	0.086 (2)	0.094 (2)	0.0098 (13)	0.0173 (14)	0.0046 (17)
C19	0.0522 (14)	0.106 (2)	0.0698 (16)	0.0003 (14)	0.0069 (12)	0.0066 (17)
C20	0.0617 (15)	0.107 (2)	0.0510 (13)	−0.0126 (14)	0.0024 (11)	−0.0092 (14)
C21	0.0571 (13)	0.0837 (18)	0.0520 (12)	−0.0119 (12)	0.0031 (10)	−0.0120 (12)
C22	0.0502 (12)	0.0702 (15)	0.0426 (11)	−0.0049 (10)	0.0026 (9)	0.0059 (10)
C23	0.0472 (13)	0.121 (2)	0.0422 (11)	−0.0032 (13)	−0.0024 (9)	−0.0094 (13)
C24	0.0551 (14)	0.103 (2)	0.0562 (13)	0.0078 (13)	0.0047 (11)	−0.0046 (13)
C25	0.0454 (12)	0.0638 (16)	0.0845 (17)	0.0013 (11)	−0.0013 (11)	0.0055 (13)
C26	0.0532 (14)	0.0749 (18)	0.0866 (18)	−0.0057 (12)	−0.0158 (12)	−0.0094 (15)
C27	0.0624 (14)	0.0667 (16)	0.0550 (13)	−0.0067 (11)	−0.0054 (11)	−0.0054 (11)
C28	0.086 (2)	0.155 (3)	0.093 (2)	0.019 (2)	0.0118 (17)	0.037 (2)
C29	0.087 (2)	0.102 (2)	0.104 (2)	−0.0038 (16)	0.0286 (16)	−0.0380 (19)
C30	0.0531 (15)	0.098 (2)	0.139 (3)	0.0041 (14)	0.0099 (16)	−0.015 (2)

### Geometric parameters (Å, °)

**Table:**

S1—O1	1.4271 (15)	S2—O4	1.434 (2)
S1—O2	1.4415 (14)	S2—O3	1.4352 (16)
S1—N1	1.6287 (17)	S2—N2	1.6184 (19)
S1—C1	1.761 (2)	S2—C16	1.773 (2)
N1—C7	1.424 (2)	N2—C22	1.416 (3)
N1—H1N	0.849 (9)	N2—H2N	0.852 (10)
C1—C6	1.394 (3)	C16—C21	1.393 (3)
C1—C2	1.398 (3)	C16—C17	1.395 (3)
C2—C3	1.394 (3)	C17—C18	1.398 (4)
C2—C13	1.507 (3)	C17—C29	1.513 (3)
C3—C4	1.384 (3)	C18—C19	1.383 (4)
C3—H3	0.9300	C18—H18	0.9300
C4—C5	1.382 (3)	C19—C20	1.375 (4)
C4—C14	1.513 (3)	C19—C28	1.511 (4)
C5—C6	1.374 (3)	C20—C21	1.387 (3)
C5—H5	0.9300	C20—H20	0.9300
C6—H6	0.9300	C21—H21	0.9300
C7—C8	1.372 (3)	C22—C27	1.378 (3)
C7—C12	1.382 (3)	C22—C23	1.378 (3)
C8—C9	1.379 (3)	C23—C24	1.382 (3)
C8—H8	0.9300	C23—H23	0.9300
C9—C10	1.377 (3)	C24—C25	1.366 (3)
C9—H9	0.9300	C24—H24	0.9300
C10—C11	1.377 (3)	C25—C26	1.384 (4)
C10—C15	1.513 (3)	C25—C30	1.514 (3)
C11—C12	1.383 (3)	C26—C27	1.381 (3)
C11—H11	0.9300	C26—H26	0.9300
C12—H12	0.9300	C27—H27	0.9300
C13—H13A	0.9600	C28—H28A	0.9600
C13—H13B	0.9600	C28—H28B	0.9600
C13—H13C	0.9600	C28—H28C	0.9600
C14—H14A	0.9600	C29—H29A	0.9600
C14—H14B	0.9600	C29—H29B	0.9600
C14—H14C	0.9600	C29—H29C	0.9600
C15—H15A	0.9600	C30—H30A	0.9600
C15—H15B	0.9600	C30—H30B	0.9600
C15—H15C	0.9600	C30—H30C	0.9600
			
O1—S1—O2	117.90 (9)	O4—S2—O3	118.92 (11)
O1—S1—N1	108.88 (10)	O4—S2—N2	109.47 (12)
O2—S1—N1	104.38 (9)	O3—S2—N2	104.20 (10)
O1—S1—C1	107.68 (9)	O4—S2—C16	107.45 (11)
O2—S1—C1	110.01 (9)	O3—S2—C16	108.68 (11)
N1—S1—C1	107.55 (9)	N2—S2—C16	107.65 (10)
C7—N1—S1	124.81 (13)	C22—N2—S2	128.39 (16)
C7—N1—H1N	116.7 (16)	C22—N2—H2N	118.2 (18)
S1—N1—H1N	109.5 (15)	S2—N2—H2N	113.3 (18)
C6—C1—C2	120.94 (19)	C21—C16—C17	120.3 (2)
C6—C1—S1	116.52 (16)	C21—C16—S2	116.5 (2)
C2—C1—S1	122.53 (15)	C17—C16—S2	123.18 (18)
C3—C2—C1	116.63 (19)	C16—C17—C18	117.1 (2)
C3—C2—C13	118.7 (2)	C16—C17—C29	123.6 (3)
C1—C2—C13	124.6 (2)	C18—C17—C29	119.2 (3)
C4—C3—C2	123.6 (2)	C19—C18—C17	123.3 (3)
C4—C3—H3	118.2	C19—C18—H18	118.4
C2—C3—H3	118.2	C17—C18—H18	118.4
C5—C4—C3	117.6 (2)	C20—C19—C18	118.0 (3)
C5—C4—C14	121.6 (2)	C20—C19—C28	120.5 (3)
C3—C4—C14	120.8 (2)	C18—C19—C28	121.4 (3)
C6—C5—C4	121.4 (2)	C19—C20—C21	121.0 (2)
C6—C5—H5	119.3	C19—C20—H20	119.5
C4—C5—H5	119.3	C21—C20—H20	119.5
C5—C6—C1	119.8 (2)	C20—C21—C16	120.2 (3)
C5—C6—H6	120.1	C20—C21—H21	119.9
C1—C6—H6	120.1	C16—C21—H21	119.9
C8—C7—C12	118.81 (19)	C27—C22—C23	118.9 (2)
C8—C7—N1	117.91 (18)	C27—C22—N2	117.2 (2)
C12—C7—N1	123.26 (19)	C23—C22—N2	123.90 (19)
C7—C8—C9	120.4 (2)	C22—C23—C24	120.1 (2)
C7—C8—H8	119.8	C22—C23—H23	119.9
C9—C8—H8	119.8	C24—C23—H23	119.9
C10—C9—C8	121.9 (2)	C25—C24—C23	122.2 (2)
C10—C9—H9	119.0	C25—C24—H24	118.9
C8—C9—H9	119.0	C23—C24—H24	118.9
C11—C10—C9	116.9 (2)	C24—C25—C26	116.8 (2)
C11—C10—C15	121.9 (2)	C24—C25—C30	121.3 (2)
C9—C10—C15	121.1 (2)	C26—C25—C30	121.9 (2)
C10—C11—C12	122.2 (2)	C27—C26—C25	122.3 (2)
C10—C11—H11	118.9	C27—C26—H26	118.9
C12—C11—H11	118.9	C25—C26—H26	118.9
C7—C12—C11	119.8 (2)	C22—C27—C26	119.6 (2)
C7—C12—H12	120.1	C22—C27—H27	120.2
C11—C12—H12	120.1	C26—C27—H27	120.2
C2—C13—H13A	109.5	C19—C28—H28A	109.5
C2—C13—H13B	109.5	C19—C28—H28B	109.5
H13A—C13—H13B	109.5	H28A—C28—H28B	109.5
C2—C13—H13C	109.5	C19—C28—H28C	109.5
H13A—C13—H13C	109.5	H28A—C28—H28C	109.5
H13B—C13—H13C	109.5	H28B—C28—H28C	109.5
C4—C14—H14A	109.5	C17—C29—H29A	109.5
C4—C14—H14B	109.5	C17—C29—H29B	109.5
H14A—C14—H14B	109.5	H29A—C29—H29B	109.5
C4—C14—H14C	109.5	C17—C29—H29C	109.5
H14A—C14—H14C	109.5	H29A—C29—H29C	109.5
H14B—C14—H14C	109.5	H29B—C29—H29C	109.5
C10—C15—H15A	109.5	C25—C30—H30A	109.5
C10—C15—H15B	109.5	C25—C30—H30B	109.5
H15A—C15—H15B	109.5	H30A—C30—H30B	109.5
C10—C15—H15C	109.5	C25—C30—H30C	109.5
H15A—C15—H15C	109.5	H30A—C30—H30C	109.5
H15B—C15—H15C	109.5	H30B—C30—H30C	109.5
			
O1—S1—N1—C7	−68.1 (2)	O4—S2—N2—C22	40.9 (2)
O2—S1—N1—C7	165.14 (18)	O3—S2—N2—C22	169.1 (2)
C1—S1—N1—C7	48.3 (2)	C16—S2—N2—C22	−75.6 (2)
O1—S1—C1—C6	−3.23 (17)	O4—S2—C16—C21	−1.54 (19)
O2—S1—C1—C6	126.48 (15)	O3—S2—C16—C21	−131.43 (17)
N1—S1—C1—C6	−120.42 (15)	N2—S2—C16—C21	116.28 (17)
O1—S1—C1—C2	177.66 (15)	O4—S2—C16—C17	177.29 (17)
O2—S1—C1—C2	−52.64 (18)	O3—S2—C16—C17	47.4 (2)
N1—S1—C1—C2	60.47 (18)	N2—S2—C16—C17	−64.9 (2)
C6—C1—C2—C3	0.9 (3)	C21—C16—C17—C18	−0.5 (3)
S1—C1—C2—C3	179.96 (14)	S2—C16—C17—C18	−179.28 (16)
C6—C1—C2—C13	−179.9 (2)	C21—C16—C17—C29	179.3 (2)
S1—C1—C2—C13	−0.8 (3)	S2—C16—C17—C29	0.5 (3)
C1—C2—C3—C4	0.3 (3)	C16—C17—C18—C19	0.3 (4)
C13—C2—C3—C4	−179.0 (2)	C29—C17—C18—C19	−179.5 (2)
C2—C3—C4—C5	−1.7 (3)	C17—C18—C19—C20	0.2 (4)
C2—C3—C4—C14	176.7 (2)	C17—C18—C19—C28	−178.0 (2)
C3—C4—C5—C6	1.8 (3)	C18—C19—C20—C21	−0.5 (4)
C14—C4—C5—C6	−176.6 (2)	C28—C19—C20—C21	177.7 (2)
C4—C5—C6—C1	−0.6 (3)	C19—C20—C21—C16	0.3 (3)
C2—C1—C6—C5	−0.8 (3)	C17—C16—C21—C20	0.2 (3)
S1—C1—C6—C5	−179.88 (16)	S2—C16—C21—C20	179.10 (17)
S1—N1—C7—C8	−143.38 (19)	S2—N2—C22—C27	−168.06 (19)
S1—N1—C7—C12	38.0 (3)	S2—N2—C22—C23	12.9 (4)
C12—C7—C8—C9	0.6 (3)	C27—C22—C23—C24	−1.7 (4)
N1—C7—C8—C9	−178.1 (2)	N2—C22—C23—C24	177.3 (2)
C7—C8—C9—C10	−0.9 (4)	C22—C23—C24—C25	−0.1 (4)
C8—C9—C10—C11	0.7 (4)	C23—C24—C25—C26	1.7 (4)
C8—C9—C10—C15	−178.9 (3)	C23—C24—C25—C30	−177.8 (3)
C9—C10—C11—C12	−0.4 (4)	C24—C25—C26—C27	−1.5 (4)
C15—C10—C11—C12	179.2 (2)	C30—C25—C26—C27	178.0 (2)
C8—C7—C12—C11	−0.3 (3)	C23—C22—C27—C26	1.9 (4)
N1—C7—C12—C11	178.3 (2)	N2—C22—C27—C26	−177.2 (2)
C10—C11—C12—C7	0.2 (3)	C25—C26—C27—C22	−0.3 (4)

### Hydrogen-bond geometry (Å, °)

**Table:**

*D*—H···*A*	*D*—H	H···*A*	*D*···*A*	*D*—H···*A*
N1—H1N···O3^i^	0.85 (1)	2.15 (1)	2.991 (2)	169 (2)
N2—H2N···O2^ii^	0.85 (1)	2.03 (1)	2.877 (2)	175 (3)

Symmetry codes: (i) −*x*+1, *y*+1/2, −*z*+1/2; (ii) −*x*+1, *y*−1/2, −*z*+1/2.
